# Uncovering full-length transcript isoforms of sugarcane cultivar Khon Kaen 3 using single-molecule long-read sequencing

**DOI:** 10.7717/peerj.5818

**Published:** 2018-10-30

**Authors:** Jittima Piriyapongsa, Pavita Kaewprommal, Sirintra Vaiwsri, Songtham Anuntakarun, Warodom Wirojsirasak, Prapat Punpee, Peeraya Klomsa-ard, Philip J. Shaw, Wirulda Pootakham, Thippawan Yoocha, Duangjai Sangsrakru, Sithichoke Tangphatsornruang, Sissades Tongsima, Somvong Tragoonrung

**Affiliations:** 1 National Center for Genetic Engineering and Biotechnology (BIOTEC), National Science and Technology Development Agency (NSTDA), Pathum Thani, Thailand; 2 Mitr Phol Sugarcane Research Center Co., Ltd., Chaiyaphum, Thailand

**Keywords:** Sugarcane, Full-length transcripts, Transcriptome, Single-molecule long-read sequencing, Iso-Seq, PacBio sequencing, Khon Kaen 3, KK3

## Abstract

**Background:**

Sugarcane is an important global food crop and energy resource. To facilitate the sugarcane improvement program, genome and gene information are important for studying traits at the molecular level. Most currently available transcriptome data for sugarcane were generated using second-generation sequencing platforms, which provide short reads. The *de novo* assembled transcripts from these data are limited in length, and hence may be incomplete and inaccurate, especially for long RNAs.

**Methods:**

We generated a transcriptome dataset of leaf tissue from a commercial Thai sugarcane cultivar Khon Kaen 3 (KK3) using PacBio RS II single-molecule long-read sequencing by the Iso-Seq method. Short-read RNA-Seq data were generated from the same RNA sample using the Ion Proton platform for reducing base calling errors.

**Results:**

A total of 119,339 error-corrected transcripts were generated with the N50 length of 3,611 bp, which is on average longer than any previously reported sugarcane transcriptome dataset. 110,253 sequences (92.4%) contain an open reading frame (ORF) of at least 300 bp long with ORF N50 of 1,416 bp. The mean lengths of 5′ and 3′ untranslated regions in 73,795 sequences with complete ORFs are 1,249 and 1,187 bp, respectively. 4,774 transcripts are putatively novel full-length transcripts which do not match with a previous Iso-Seq study of sugarcane. We annotated the functions of 68,962 putative full-length transcripts with at least 90% coverage when compared with homologous protein coding sequences in other plants.

**Discussion:**

The new catalog of transcripts will be useful for genome annotation, identification of splicing variants, SNP identification, and other research pertaining to the sugarcane improvement program. The putatively novel transcripts suggest unique features of KK3, although more data from different tissues and stages of development are needed to establish a reference transcriptome of this cultivar.

## Introduction

Sugarcane (*Saccharum officinarum* L.) is one of the most efficient biomass producers (Vermerris, 2011) and an economically important crop. About 75% of sugar for food consumption worldwide is produced from sugarcane (Commodity Research Bureau, 2015). Moreover, the sugar extraction process from sugarcane generates a large quantity of bagasse by-product, and this lignocellulosic biomass is recognized as a future feedstock for ethanol production (Dias et al., 2009).

Modern sugarcane cultivars are hybrids derived from interspecific crosses about a century ago between *Saccharum officinarum* and *Saccharum spontaneum.* They have large, highly polyploid and aneuploid genomes, consisting of 100 to 130 chromosomes. 70–80% of the sugarcane genome is from *S. officinarum*, 10–20% from *S. spontaneum*, and about 10% from recombinations of these two species (D’Hont, 2005; D’Hont et al., 1996; D’Hont et al., 2008). The daunting size and complexity of the sugarcane genome has hindered genomic research in the organism. Draft reference genome sequences are available, with the most recent providing 393x monoploid coverage of the SP80-3280 cultivar (Riaño Pachón & Mattiello, 2017). However, a finished sequence of the full chromosome complement is elusive (Thirugnanasambandam, Hoang & Henry, 2018). Furthermore, the high heterozygosity and chromosomal variation among cultivars means that a reference genome for one cultivar partially represents that of others.

The lack of adequate genome resources for sugarcane mean that transcriptome data are vital for identifying genes and studying their functions related to economically important traits such as sugar content and stress tolerance (Manners & Casu, 2011). Moreover, transcriptome data can provide insights into gene content and help future genome annotation. Previous studies of the sugarcane transcriptome have employed expressed sequence tag (EST) sequencing, and more recently Next Generation Sequencing (NGS). NGS has been applied for sugarcane transcriptomic study using second-generation sequencing platforms (Cardoso-Silva et al., 2014; Dharshini et al., 2016; Huang et al., 2016; Li et al., 2016; Nishiyama Jr et al., 2014; Schaker et al., 2016; Vicentini et al., 2015). Second-generation sequencing of RNA (RNA-Seq) produces short sequence reads (<1,000 nt). Transcript sequences must be reconstructed from RNA-Seq data by the process of *de novo* assembly, in which overlapping reads are identified. For organisms which lack a reference genome, *de novo* transcriptome assembly of short reads is inaccurate, especially for polyploid organisms with homo(eo)logous gene sets consisting of nearly identical gene sequences.

Single-molecule, real time (SMRT) sequencing technology developed by Pacific Biosciences (PacBio) can provide much longer (kilobase-sized) reads than second-generation platforms (Eid et al., 2009). Reads spanning full-length transcripts from the polyA-tail to the 5′ end can be obtained from SMRT sequencing of cDNA libraries in the so-called Isoform Sequencing (Iso-Seq) protocol. In addition to eliminating the need for error-prone assembly of short sequence reads, Iso-Seq provides more insight into transcript isoform complexity than RNA-Seq (Au et al., 2013). This is advantageous especially for organisms which lack a high-quality reference genome like sugarcane. A recent Iso-Seq study of sugarcane reported 107,598 transcript isoforms of a pooled RNA sample derived from leaf, internode and root tissues of different developmental stages from 22 varieties (Hoang et al., 2017). Although this transcript isoform catalog is important for sugarcane genome annotation, the pooling of samples possibly obscured transcripts unique to certain tissues or cultivars. Transcripts unique to certain cultivars could associate with agronomic traits and thus provide new markers for breed improvement programs. Iso-Seq studies of individual cultivars are thus warranted.

Thailand is the fourth largest producer and second largest exporter of sugarcane in the world (USDA Foreign Agricultural Service, 2017). Khon Kaen 3 (KK3) is the most widely planted commercial variety in Thailand, at around 60–70% of cultivatable area. KK3 is an offspring of clone 85-2-352 (female) and K84-200 (male) (Tippayawat, Ponragdee & Sansayawichai, 2012). This cultivar is non-flowering, drought-tolerant, produces high yield (100–112.5 tonnes/ha), sugar (14–15% Commercial Cane Sugar) and stalk number (68,750–75,000 stalks/ha), exhibits good ratooning, and is suitable for mechanical harvesting (Department of Agriculture Thailand, 2018). Previous transcriptome studies did not include the KK3 cultivar.

In this study, we obtained transcriptome data from leaf tissue of KK3 using the Iso-Seq protocol with PacBio SMRT long read technology. The majority of transcript isoforms identified in the data matched sequences in other plant and sugarcane databases. Furthermore, we discovered several new transcripts coding for novel proteins and a number of putative long non-coding RNAs.

## Materials and Methods

### Sugarcane sampling, RNA extraction, and cDNA library construction

A leaf sample was collected from a 3-month old plant of the commercial sugarcane cultivar Khon Kaen 3 (Tippayawat, Ponragdee & Sansayawichai, 2012). The plant was cultivated at the National Center for Genetic Engineering and Biotechnology, National Science and Technology Development Agency, Thailand. No specific permits were required and no protected species were involved in this study. Young leaf (3 g) was collected for total RNA extraction using a QIAGEN RNeasy^®^ Mini Kit. Total RNA was treated with DNaseI (DNA-free™ kit; Ambion, Foster City, CA, USA) to remove genomic DNA contamination. From 100 µg of total RNA, mRNA (600 ng) was isolated using an Absolutely mRNA Purification kit (Agilent Technologies, Santa Clara, CA, USA).

PacBio Iso-Seq libraries were prepared according to the Isoform Sequencing (Iso-Seq) protocol using the Clontech SMARTer PCR cDNA Synthesis Kit and the BluePippin Size Selection System protocol. In brief, a 35 ng sample of mRNA was reverse-transcribed to synthesize first-strand cDNA. The first-strand cDNA was amplified for 12 PCR cycles using KAPA HiFi DNA polymerase (Kapa Biosystems, Wilmington, MA, USA). cDNA was size-selected into 4 bins of size ranges 1–2 kb, 2–3 kb, 3–6 kb and 5–10 kb using a BluePippin Size-Selection system (Sage Science, Beverly, MA, USA). cDNA from each bin was amplified for 15 PCR cycles, using the following cycles: 95 °C for 2 min follows by 15 cycles of 98 °C for 20 s, 65 °C for 15 s, 72 °C for 4 min and a final extension of 72 °C for 5 min. The amplified DNA was subjected to damage and end-repair, and then ligated to a SMRTbell adapter for Iso-Seq SMRTbell library construction. Before sequencing, a sequencing primer was annealed to SMRTbell templates which were then bound to DNA polymerase and prepared for Magbead loading onto SMRT cells. Sequencing was performed on a PacBio RS II system using P6C4 polymerase and chemistry and a 240-min movie time. Libraries composed of transcripts in the size ranges 1–2 kb, 2–3 kb, and 3–6 kb were each sequenced on two cells and transcripts of size 5–10 kb were sequenced on one cell.

100 µg of purified mRNA from the same leaf sample was used to construct an RNA-Seq library using the Ion Total RNA-Seq Kit v2 (Thermo Fisher Scientific, Waltham, MA, USA) according to the manufacturer’s instruction. The mRNA was fragmented using RNaseIII (Thermo Fisher Scientific, Waltham, MA, USA) to obtain fragments with a mean length of around 200 nt. Subsequently, adapters were ligated to fragmented mRNA and first strand cDNA synthesis was performed using SuperScript™ III (Thermo Fisher Scientific, Waltham, MA, USA). The DNA library was sequenced on the Ion Proton using Ion PI™ Chip Kit v3 chips (Thermo Fisher Scientific, Waltham, MA, USA).

### Sequencing data processing, isoform clustering and error correction

The raw reads generated from SMRT sequencing were processed separately for each size-selected library using the “RS_IsoSeq” protocol (https://github.com/PacificBiosciences/cDNA_primer/wiki) (Gordon et al., 2015) available in SMRT^®^ Analysis software v2.3.0 (http://www.pacb.com/support/software-downloads), a free software suite that can be downloaded from the Pacific Biosciences website. The analysis started by generating read of insert (ROI), the representative single sequence of highest quality for an insert generated by self-correction through subread comparison. The ROIs were then classified into full-length non-chimeric and non-full-length reads. The full-length reads with minimum length of 300 bp were clustered and used to construct consensus isoform sequences by the ICE (isoform-level clustering) algorithm. These consensus sequences were then polished using the Quiver algorithm to get high quality isoform sequences with at least 99% accuracy. High quality and low quality isoform sequences from all size fractions were combined into a final transcript sequence dataset for subsequent analyses. To improve the quality of polished PacBio transcripts, short-read error correction was performed on unique transcript isoforms using Ion Proton reads sequenced from the same RNA sample by using LoRDEC software version 0.6 with parameters of -k 21 -s 3 -t 5 -b 200 -e 0.4. Ion proton reads used for correction were preprocessed by sequence trimming of poor-quality base calls (N) and removing reads with ambiguous bases, reads of low overall quality (average Phred quality score < 17), and reads shorter than 50 bp using PRINSEQ version 0.20.4 (Schmieder & Edwards, 2011). BLASTN search against Rfam database version 12.2 (Nawrocki et al., 2015) was performed to remove rRNA sequences from the preprocessed Ion Proton reads before PacBio error correction process. Redundant sequences in the corrected transcripts were removed using CD-HIT (Fu et al., 2012; Li & Godzik, 2006) with 99% sequence identity cutoff. PacBio and Ion Proton raw read data were deposited in the NCBI Sequence Read Archive (SRA) database under accession number SRP103465.

### Prediction of coding regions within transcripts

The identification of open reading frame (ORF) within transcript sequences was performed using Transdecoder software version 3.0.1 (Haas et al., 2013) (https://transdecoder.github.io). A Markov model was trained using the top longest ORFs initially identified across all transcripts. The software then identified candidate protein-coding regions on each transcript based on log-likelihood scores calculated from this model. Homology based on BLASTP (version ncbi-blast-2.6.0+) (Altschul et al., 1990) results of all translated ORFs against Phytozome version 12 (Goodstein et al., 2012) plant proteins and the presence of protein domain(s) from the Pfam database (version 31.0) (Finn et al., 2016) were incorporated as additional criteria for ORF prediction. The parameter settings for BLASTP and Pfam were as shown in the Transdecoder GitHub (https://transdecoder.github.io). A single best ORF of minimum length 300 bp was finally selected as the predicted ORF for each transcript. Protein domains in the assigned ORF of constructed transcripts were predicted using InterProScan 5.22–61.0 with default settings which search against protein domains/signatures from a number of different databases (Jones et al., 2014).

### Functional annotation and similarity matching of PacBio isoforms

Sequence similarity search was performed on PacBio transcripts against various nucleotide and protein sequence databases using BLASTN and BLASTX (version blast-2.2.26), respectively (Altschul et al., 1990) with an *E*-value cutoff of 10^−6^. The match was assigned to the first hit with the highest bit score. Homology search among previously described sugarcane sequences was performed using the PlantGDB version 157a (http://www.plantgdb.org), SoGI release 3 (http://compbio.dfci.harvard.edu/tgi/) and NCBI dbEST release 130101 (Boguski, Lowe & Tolstoshev, 1993). Homology search among other plant sequences was conducted using the Phytozome version 12 database (Goodstein et al., 2012). Other homologous functions were annotated by searching the NCBI non-redundant protein database (nr) (downloaded in August 2016). Finally, sequence similarity search was performed on PacBio transcripts against long non-coding RNA (lncRNA) sequences downloaded from lncRNAdb v2.0 (Amaral et al., 2011), CANTATAdb v1.0 (Szczesniak, Rosikiewicz & Makalowska, 2016), NONCODE 2016 (Zhao et al., 2016) and Plant non-coding RNA Database (PNRD) (Yi et al., 2015).

To identify PacBio transcripts corresponding to putatively full-length transcripts spanning protein coding sequences (CDS), BLASTN search results of PacBio transcripts against plant CDS of Phytozome version 12 database (Goodstein et al., 2012) were used for calculation of length coverage of PacBio transcripts on the corresponding plant CDS with the highest bit score. Full-length transcripts were defined as PacBio transcripts with at least 90% coverage on matched plant CDS. The length ratios of PacBio transcripts to matched sequences from sugarcane databases (PlantGDB, NCBI dbEST, SoGI), PacBio transcripts from the SUGIT database (Hoang et al., 2017), and Phytozome plant transcript version 12 database were also calculated.

Gene identifiers (e.g., gene names, Gene Ontology (GO) terms, E.C. number, KOG ID and KEGG ID) were assigned to each PacBio transcript based on the hit with the highest bit score from BLASTN search against the Phytozome version 12 plant transcript sequences. The assigned GO terms were summarized in three categories, namely cellular component, molecular function, and biological process, using WEGO software (Ye et al., 2006). The assigned KOG ID is the eukaryotic orthologous group (KOG) which was constructed based on orthologous and paralogous proteins from different eukaryotic genomes to infer possible function of transcript, information of which is available in NCBI COG database (https://www.ncbi.nlm.nih.gov/COG) (Tatusov et al., 2003). To determine metabolic and biological pathways that are related to transcript sequences, KEGG Orthology (KO) associated with orthologous proteins was used in order to link to KEGG pathways (Kanehisa & Goto, 2000; Kanehisa et al., 2016). Only plant-related pathways of the KEGG database were applied.

PacBio isoforms from this study were matched with isoforms in the SUGIT database (Hoang et al., 2017) by BLASTN (version blast-2.2.26) (Altschul et al., 1990) analysis with an *E*-value cutoff of 10^−20^.

### Prediction of alternatively spliced transcripts

PacBio isoforms from this study and in the SUGIT database (Hoang et al., 2017) were aligned against the sorghum genome (Sbicolor_454_v3.0.1) from Phytozome version 12 using GMAP software (Wu & Watanabe, 2005) version 2015-06-23 with the parameter settings recommended for alignment of PacBio Iso-Seq transcripts to the genome (—no-chimeras—cross-species -expand -offsets 1 -B 5 -K 8000 -f samse -n 1 -t 8—min-trimmed-coverage=0.9 -min-identity=0.8) provided in the TAPIS (Transcriptome Analysis Pipeline from Isoform Sequencing) Bitbucket repository (https://bitbucket.org/comp_bio/tapis). The potential alternative splicing events of the PacBio constructed transcripts were predicted using TAPIS software version 1.2.1 (Abdel-Ghany et al., 2016) with default settings. The alternative splicing patterns of each gene was displayed using SpliceGrapher included in the TAPIS softtware.

### Repeat sequence analysis

Repetitive sequences on PacBio transcripts were predicted using RepeatMasker version open-4.0.5 (Smit, Hubley & Green, 2013–2015) with default parameters. The transcripts were compared with classified sequences in “grasrep.ref.classified.fa”, which is a database that includes all grass family repeat sequences from RepBase Update 20140131 (Jurka et al., 2005) and RM database version 20140131.

### Gene expression analysis

The preprocessed Ion Proton reads were mapped to PacBio transcripts using TMAP software (https://github.com/iontorrent/TMAP) with the ‘mapall’ parameter and output filtered for all best hits (-a 2). The output of TMAP in BAM formatted file was used for calculation of transcript expression levels (RPKM; Reads Per Kilobase of transcript per Million mapped reads) using tigar2 software (Nariai et al., 2014) with default parameters. The transcript sequences were classified into three groups based on expression levels: low (≥0.5 to 5 RPKM), medium (≥5 to 50 RPKM) and high (≥ 50 RPKM). Gene name and KOG ID were assigned to PacBio transcripts based on the top hit from BLASTN search against the Phytozome version 12 plant transcript sequence database.

## Results

### Identification of sugarcane KK3 transcript isoforms from Iso-Seq data

PacBio transcriptome sequencing from a cDNA library of KK3 leaf sample produced a total of 4,159,174 reads of insert from all RNA size fractions. Out of these, 232,328 full-length, non-chimeric reads with a minimum length of 300 bp were obtained. After clustering of these full-length transcripts and polishing the consensus isoforms, 53,999 high-quality and 75,975 low-quality isoforms were generated. A total of 99,344,049 Ion Proton reads were filtered to 83,393,993 high-quality reads, which were then used for error correction of these Iso-Seq transcripts. After error correction of all 129,974 isoform sequences and removal of redundant sequences, 119,339 transcripts remained. We refer to these error-corrected, non-redundant transcript isoforms as PacBio-isoforms (Table S1). The PacBio-isoforms showed greater representation of long transcripts and ORFs than uncorrected transcripts (Table 1). The length of the PacBio-isoform sequences ranged from 307 to 26,751 bp, with N50, mean length, and L50 of 3,611 bp, 3,099 bp, and 36,456 sequences, respectively.

**Table 1:** Summary of Iso-Seq transcripts after error correction using short reads.

Statistics	Before error correction	After error correction
Number of unique transcripts	121,548	119,339
Transcript length range	307–26,595	307–26,751
N50 (all transcripts)	3,617	3,611
Number of transcripts (%) mapping to sorghum genome	70,326 (57.86%)	72,061 (60.38%)
ORF prediction (without homology information):		
Number of predicted ORFs	176,455	207,027
ORF N50	954	1,074
Number of transcripts with ORF	96,832	104,282
ORF prediction (with homology information):		
Number of predicted ORFs	224,910	255,442
ORF N50	816	951
ORF N50 (only single best ORF of each transcript)	1,161	1,416
Number of transcripts with ORF	105,252	110,253

### Annotation of PacBio-isoforms by comparison with available sequence data

**Figure 1:**
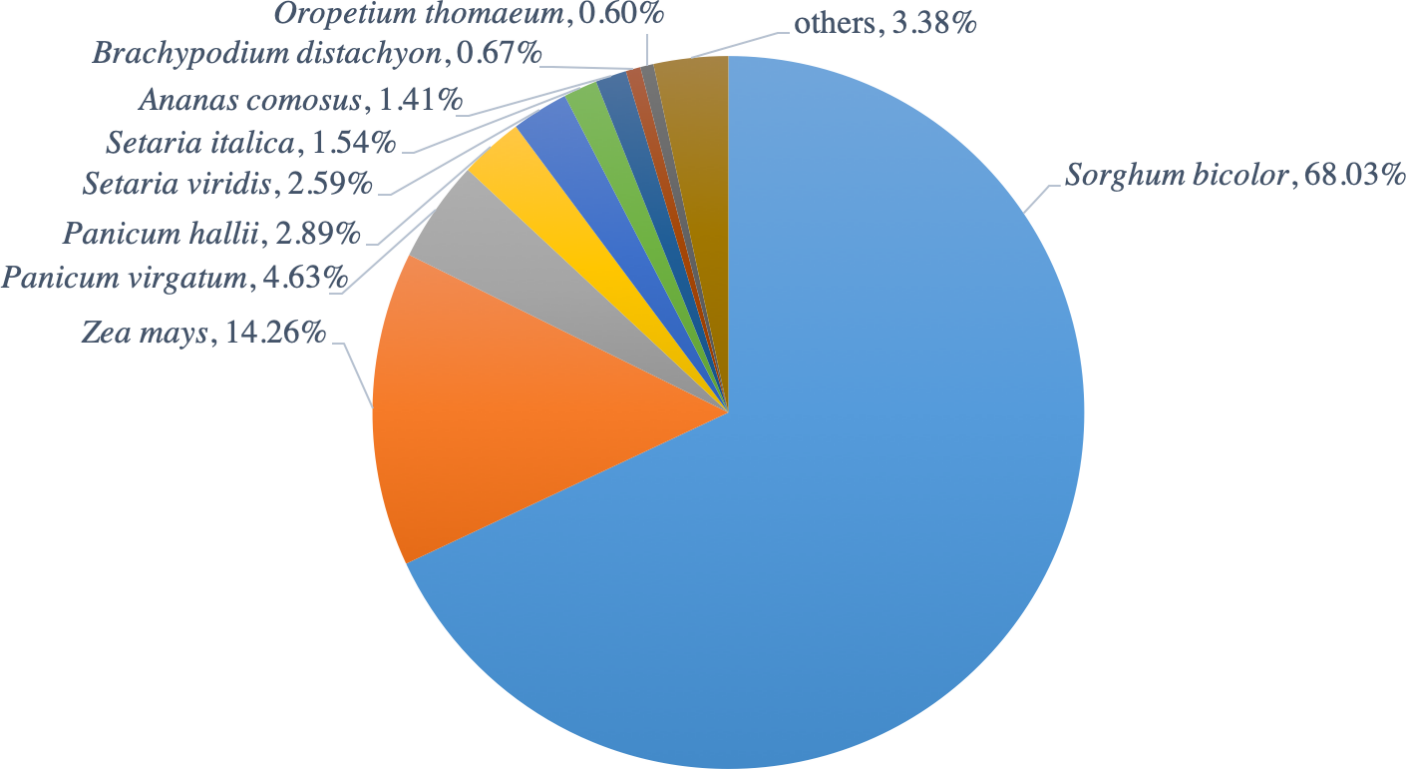
Distribution of hit plant species from BLAST search of PacBio-isoforms. Pie chart shows the fraction of hit plant species based on the best hit obtained from BLASTX search of PacBio transcripts against Phytozome plant proteins.

**Figure 2:**
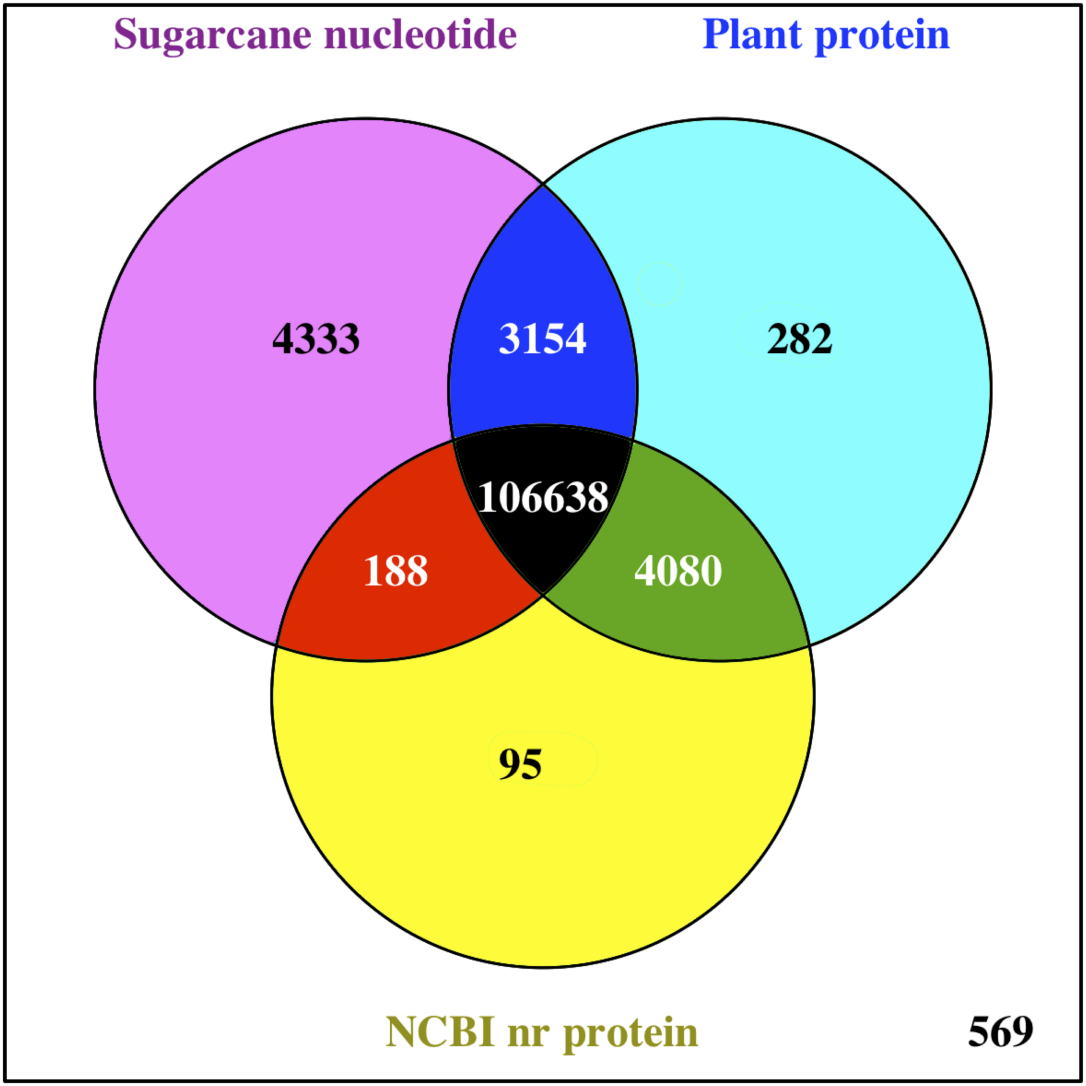
Comparison of BLAST hits from different sequence databases. Venn diagram shows overlaps of BLAST analysis results among compared databases, namely sugarcane nucleotide, Phytozome plant protein, and NCBI nr protein databases.

The functions of all PacBio-isoforms were annotated by comparison with various nucleotide and protein databases. BLAST hits in the sense direction to these databases are summarized in Table 2. A total of 107,742 sequences (90.28%) showed sequence similarity to previously described sugarcane nucleotide sequences from SoGI, NCBI EST, and PlantGDB databases. We did not include sugarcane transcript sequences in the SUGIT database (Hoang et al., 2017) for this analysis because no annotations are available for these sequences. A total of 5,026 transcript sequences did not match with any sugarcane sequence in public databases are shown in Table S2. Because sugarcane lacks a genome annotation, plant protein sequences from the Phytozome database were also used for sequence annotation. Protein matches in other plant species were found for 109,053 PacBio-isoforms (91.38%) by BLASTX analysis. The majority (68.03%) of sequences have best hits with sorghum, the species closest to sugarcane, as expected (Fig. 1). When performing the database search to the NCBI nr protein database, 95 sequences showed hits with the NCBI nr database but did not give a hit to any plant database. Most hits overlapped among sugarcane, plant protein, and nr protein databases (Fig. 2). 569 transcripts did not match with any of these three databases (Table S2, Fig. 2). A total of 4,902 sequences did not produce any significant hits with known protein sequences in any protein database. 11 of these sequences hit with known long non-coding RNAs (lncRNAs), suggesting that the remaining 4,891 sequences represent sugarcane proteins of unknown function or new lncRNAs.

**Table 2:** Summary of BLAST comparisons against different sequence databases.

Organism	Database	Sequence type	Number of transcripts with hits (%)
Sugarcane	SoGI, NCBI EST, PlantGDB	Transcript	107,742 (90.28%)
Plant	Phytozome	Transcript	108,649 (91.04%)
		CDS	107,371 (89.97%)
		Protein	109,053 (91.38%)
All	NCBI nr	Protein	107,684 (90.23%)
All	lncRNAdb, CANTATAdb, NONCODE, PNRD	lncRNA	29,066 (24.36%)

### Prediction of potential protein coding regions

To identify putative protein-coding sequences within the PacBio-isoforms, we used Transdecoder software to predict open reading frames (ORFs). When considering all types of predicted ORFs of at least 300 bp long, including partial ORFs missing start or stop codon(s), a total of 207,027 ORFs with an N50 of 1,074 bp were present among 104,282 PacBio-isoforms (87.38%) (Table 1). When homology to plant proteins and the presence of protein domain(s) were included as additional evidence for ORF prediction, a total of 255,442 ORFs with an N50 of 951 bp were identified in 110,253 ORF-containing transcripts (92.4%). The ORF N50 value increased to 1,416 bp when only the single best ORF of each transcript was used. Most of ORF-containing sequences (104,337; 94.63%) contain at least one protein motif/domain from various databases as identified by InterProScan. P-loop containing nucleoside triphosphate hydrolase and protein kinase were the most frequently found protein domains (Table S3).

**Figure 3:**
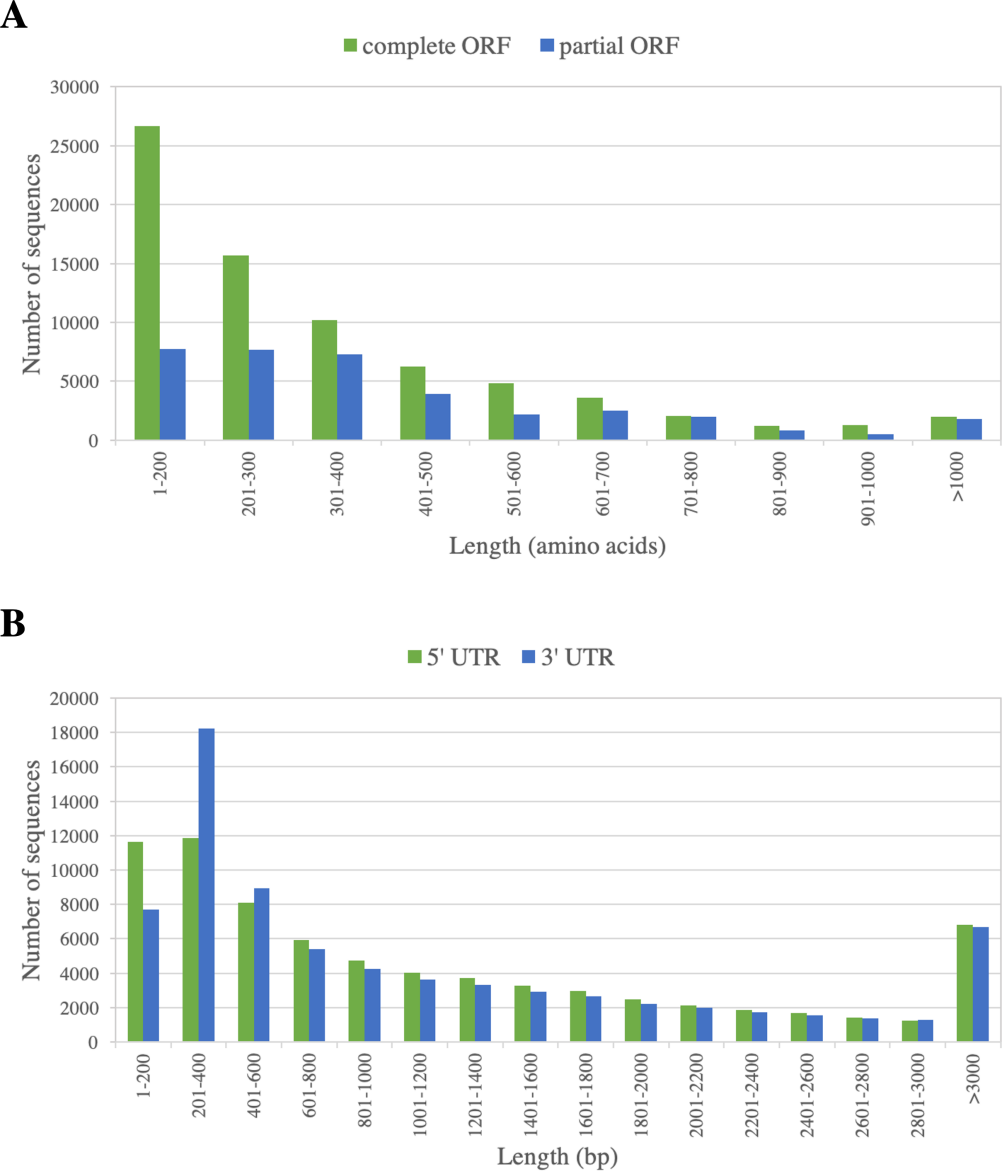
Length distribution of predicted ORFs of PacBio-isoforms. Frequency distribution graphs are displayed for (A) ORF length of complete ORFs (green) and partial ORFs (blue) and (B) UTR length calculated from the complete ORFs separated into 5′ UTR (green) and 3′ UTR (blue).

73,795 ORF-containing sequences (67%) contain a complete ORF of at least 300 bp, as indicated by the presence of start and stop codons. The ORF length varied from 300 to 6,657 bp, with the N50 of 1,344 bp. The frequency distribution of the predicted ORFs is displayed in Fig. 3. Flanking these complete ORFs, the mean lengths of 5′ and 3′ untranslated regions are 1,249 and 1,187 bp, respectively (Fig. 3).

9,086 PacBio-isoforms lacked a putative ORF longer than 300 bp. The majority of these sequences (6,558 sequences, 72.18%) have partial match with known protein sequences (from NCBI nr and/or Phytozome plant protein databases) in either sense or antisense direction. Of the remaining 2,528 sequences which did not match with known proteins, 1,219 sequences also produced no hits to Phytozome plant CDS/transcript nucleotide sequences and lack InterPro protein domain, which are suggestive of lncRNAs. Among these potential lncRNAs, only three showed significant similarity to lncRNAs in the lncRNA databases used in this study, suggesting that 1,216 sequences are putatively novel lncRNAs (Table S4). Among these putatively novel lncRNAs (average length = 2,410 bp), 173 did not contain any ORF while the remainder contain short ORFs 15 to 297 bp (median = 189 bp). Most of these transcripts are likely to be of low abundance, with average relative expression levels from Ion Proton data of 1.83 RPKM. 483 sequences did not contain any repetitive sequence or transposable element.

### Functional annotation of protein-coding transcripts

A total of 1,382 Gene Ontology (GO) terms covering three main categories (178 cellular component, 649 molecular function, and 555 biological process terms) were assigned to 72,281 PacBio-isoforms (60.57%) based on BLAST results against the Phytozome database. The distribution of GO terms found among PacBio-isoforms is displayed in Table S5. Considering the second-level GO terms, the most frequent terms for the “cellular component” category are cell (19.65%), cell part (19.65%), and membrane (18.89%). For the “molecular function” category, the majority of transcript functions are related to binding (48.07%), catalytic activity (41.78%), and transporter activity (4.70%). The top-ranked “biological process” GO terms are metabolic process (37.68%), cellular process (30.70%), and localization (7.54%). Among third-level GO terms, the most frequent terms for “cellular component”, “molecular function” and “biological process” categories are “membrane”, “protein binding”, and “protein phosphorylation”, respectively.

**Figure 4:**
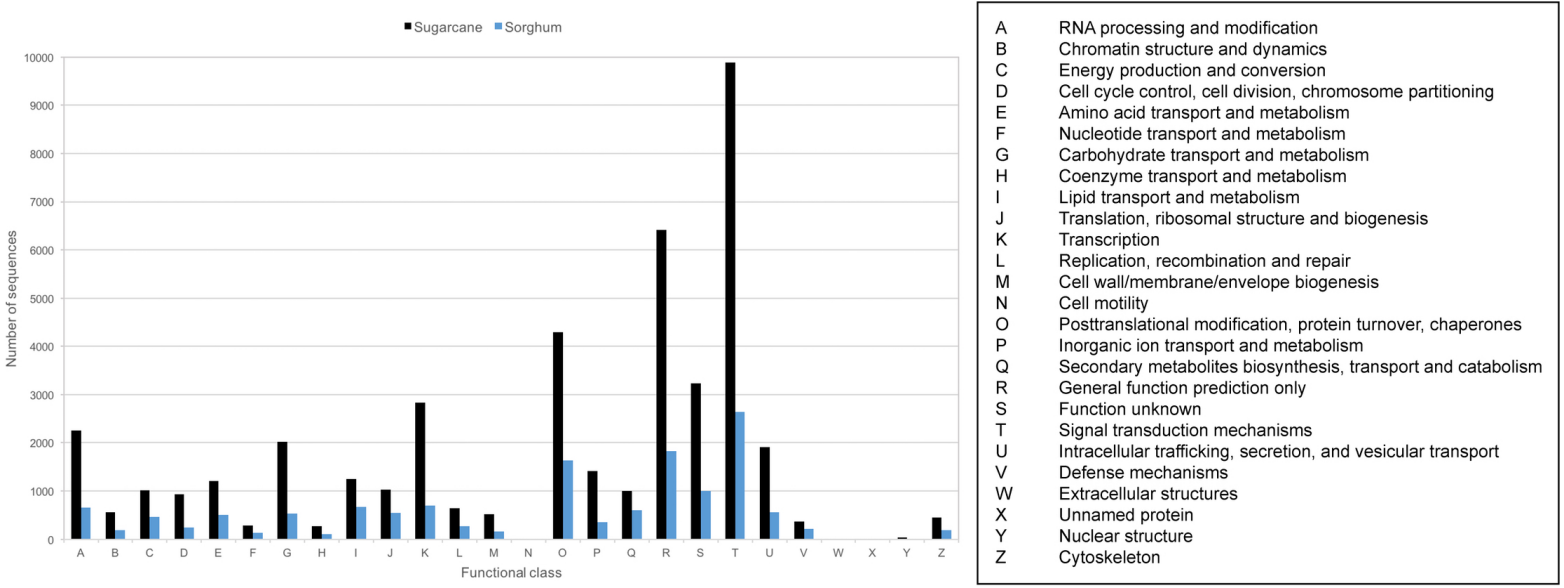
COG classification of sugarcane PacBio transcripts in comparison to sorghum transcripts. The frequency distributions of transcripts assigned to each functional class of KOG database were displayed for sugarcane PacBio transcripts (black) and sorghum transcripts available from Phytozome database (blue).

Transcript sequences were also classified to Cluster of Orthologous Groups (COG) based on groups of orthologous and paralogous proteins among eukaryotes. 43,794 transcripts (36.70%) can be assigned to 26 COG categories (Fig. 4, Table S5). The three COG categories with the highest number of related transcripts are signal transduction mechanism (22.59%), general function prediction only (14.64%), and posttranslation modification, protein turnover, chaperones (9.79%). 13,319 PacBio-isoforms were also mapped to plant-related KEGG pathways based on BLAST result search with the Phytozome database. 205 pathways were mapped (Table S5). The number of mapped transcripts per pathway ranges from 1 to 2,231 transcripts with an average of 160 transcripts per pathway. The top three pathways with the highest number of mapped transcripts are pyruvate metabolism (2,231), spliceosome (1,732), and protein processing in endoplasmic rericulum (1,342). A number of transcripts were also mapped to sugar-related pathways such as starch and sucrose metabolism (1,059), fructose and mannose metabolism (83), and plant-pathogen interaction (532).

### Analysis of repetitive elements within transcripts

We investigated repetitive sequences in PacBio-isoforms using RepeatMasker software (Smit, Hubley & Green, 2013–2015). 74,650 sequences (62.55%) contain fragments of repetitive sequences, which include interspersed repeats, small RNA, satellites, simple repeats, and low complexity sequences, while 37,758 transcript sequences (31.64%) contain fragments of interspersed repeats. The total bases masked for repetitive sequences are 6.73%, of which 5.34% correspond to interspersed repeats. 57,483 interspersed repeat elements were found, which included short interspersed nuclear elements (SINEs, 2.09%), long interspersed nuclear elements (LINEs, 13.07%), long terminal repeat elements (LTR, 24.30%), DNA transposons (46.07%), and unclassified elements (14.47%). Considering the proportion of total bases masked, LTR/Gypsy is the most represented class in the transcriptome (21.75%), followed by LTR/Copia (17.32%), LINE/L1 (13.12%), DNA/PIF-Harbinger (6.92%), and DNA/CMC-EnSpm (6.84%).

### Assessing completeness of PacBio-isoforms

**Figure 5:**
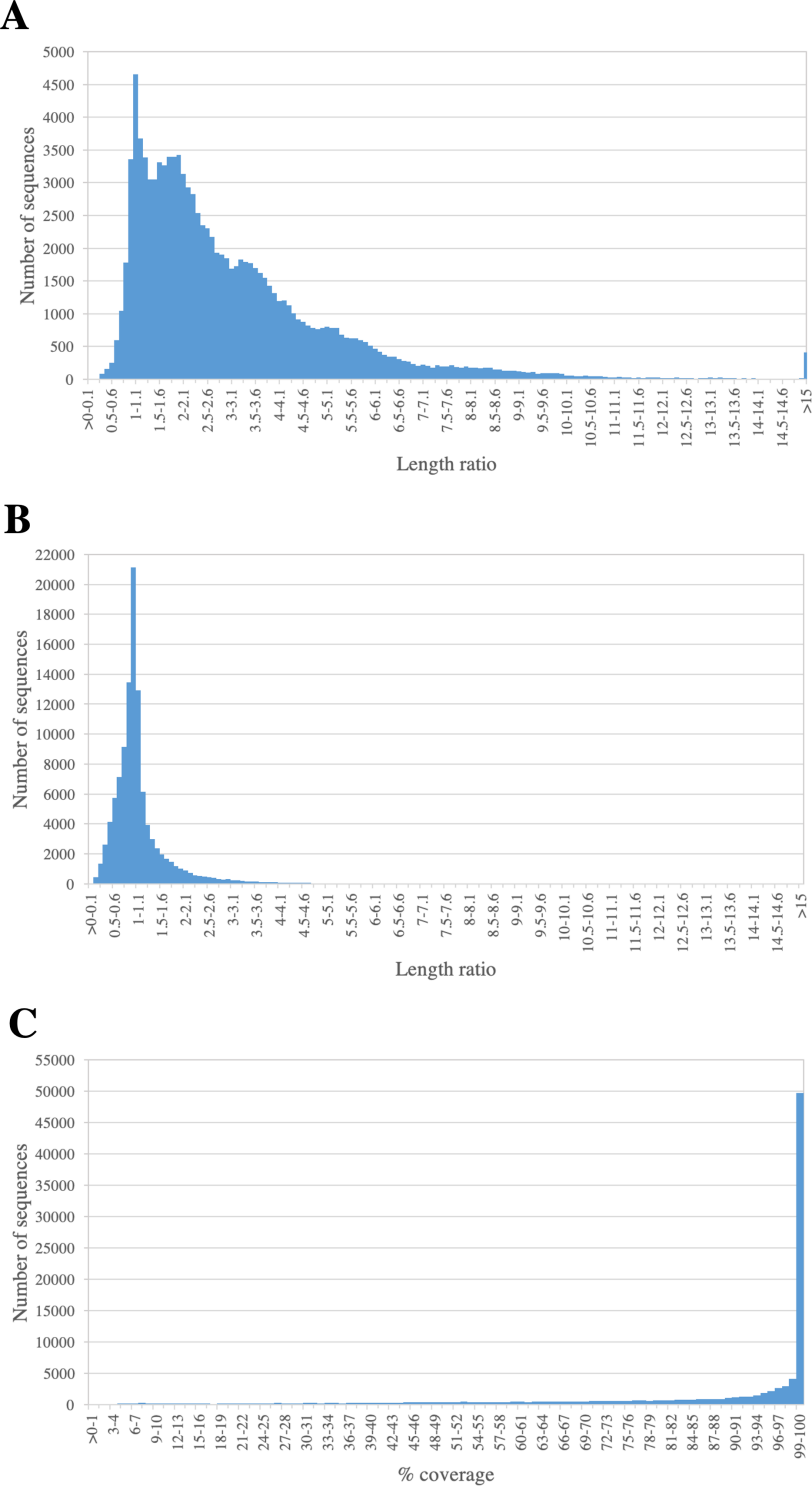
Length comparison of PacBio transcripts and their matched sequences. The graph shows the frequency distribution for the ratio of the length of PacBio transcript to its matched sequences from (A) sugarcane and (B) Phytozome plant transcript databases. The percentages of coverage on hit plant CDS sequence are shown in (C).

We assessed the completeness of PacBio-isoforms, i.e., whether they represent full-length transcripts and cover the entire CDS by comparison with related sequences in transcript databases. The length distributions of PacBio-isoforms compared with matched sugarcane and Phytozome plant sequences from BLASTN analysis are illustrated in Fig. 5. The majority (93.26%) of PacBio-isoforms are longer than matched sugarcane sequences reported in SoGI, PlantGDB, and dbEST databases. 78,095 PacBio-isoforms (71.88%) are at least 80% of the length of matched transcripts from sorghum and other plant species. We assessed the proportion of PacBio-isoforms corresponding to putatively full-length transcripts spanning CDS. Out of 107,371 PacBio-isoforms with matches in the sense direction, 68,962 (64.23%) have at least 90% coverage on matched plant CDS, suggesting that these isoforms represent full-length transcripts. Out of these full-length transcripts, 67,188 (97.43%) also contained an ORF of at least 300 bp long; however, the actual number of full-length transcripts could be higher. For sequences which have less than 90% coverage on plant CDS and sequences without plant CDS hits, 38,029 are possibly full-length transcripts of at least 1,000 bp and an ORF of at least 300 bp long.

### Comparison with previous Iso-Seq study and identification of novel sugarcane full-length transcripts

**Figure 6:**
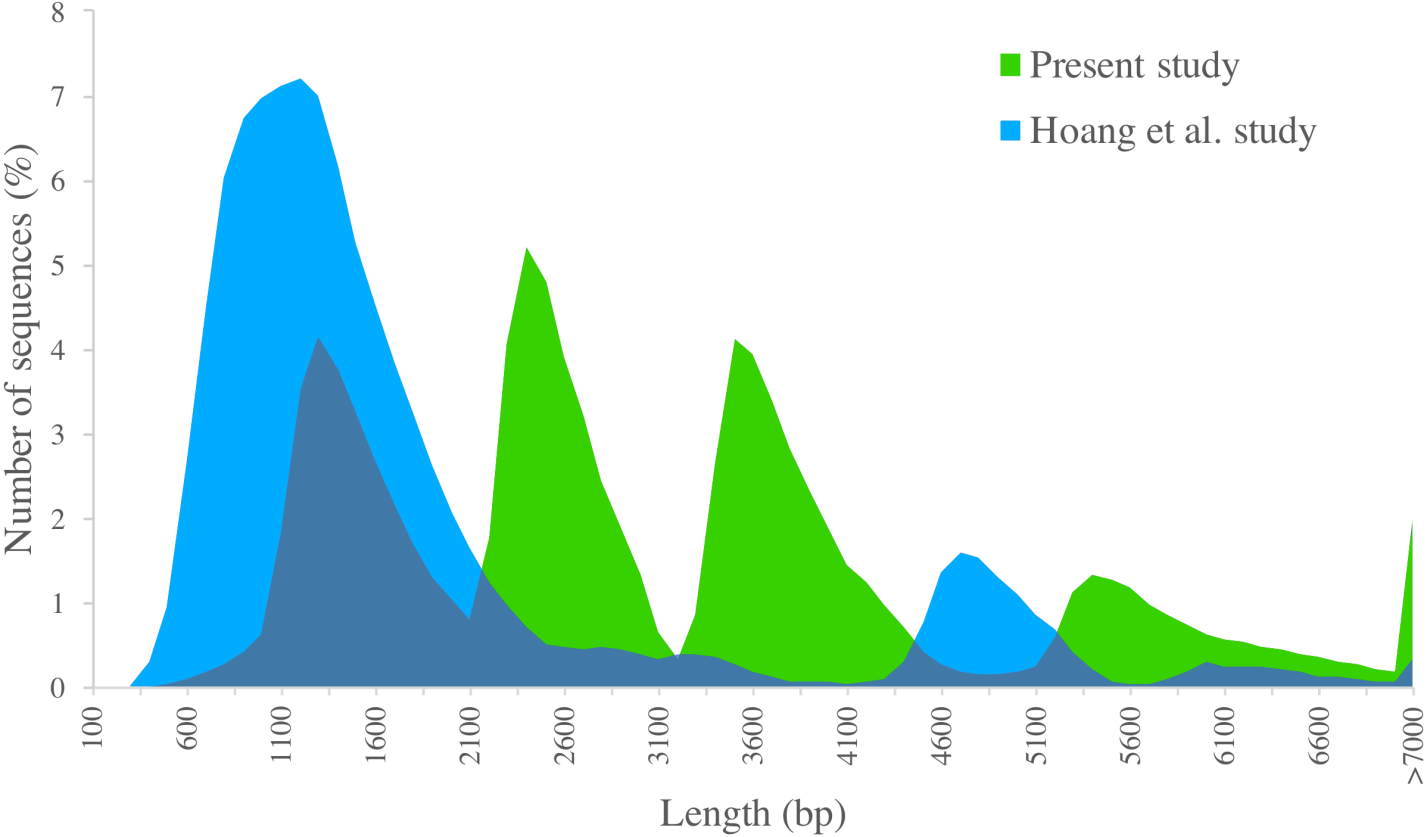
Length distribution of PacBio transcripts. Distributions of sequence length are displayed for sugarcane PacBio transcripts generated in the present study (green) and in the Hoang et al. (2017) study (blue).

We compared PacBio-isoforms with those reported in the SUGIT database (Hoang et al., 2017), which were generated using the same sequencing platform. Our study dataset is comparable in size to the SUGIT database (232,328 and 186,999 full-length non-chimeric reads, respectively), and so provides a comparable level of transcriptomic detail. While Hoang et al. explored a pooled transcriptome from various tissues and cultivars, our study was focused on the leaf tissue transcriptome of the KK3 cultivar. The frequency distributions of transcript length for our PacBio-isoforms and isoforms in the SUGIT database are displayed in Fig. 6. The PacBio-isoforms from both studies cover a broad size range including very long sequences (>7,000 bp). However, the PacBio-isoforms generated in this study are more evenly distributed across all size ranges, whereas the SUGIT PacBio-isoforms are more biased towards the size of around 1,200 bp (Fig. 6). As well as greater representation of long transcripts, our dataset contains more unique high quality transcripts than SUGIT (119,339 vs 107,598) with a greater N50 (3,611 vs 1,991 bp) and a greater number of putative full-length transcripts with at least 90% coverage of matched CDS (67,188 vs 39,999). 114,565 PacBio-isoforms (96%) match 91,683 SUGIT sequences (85.21%). 58.14% of the PacBio-isoforms are longer than the corresponding SUGIT sequence. We investigated the 4,774 PacBio-isoforms with no matching sequence in the SUGIT database in detail (Table S6). Out of these, 4,573 sequences hit with publicly available sequences (sugarcane, plant, or nr database), of which 4,163 sequences also contain an ORF. The 201 unknown sequences with no matching sequence in any database have an average length of 3,229 bp. 86 of these novel sequences contain an ORF with an average length of 3,678 bp. 64 of the ORFs are complete and thus code for putatively novel proteins. The remaining 115 novel sequences with no ORF longer than 300 bp are potentially novel lncRNAs (Tables S6, S4). From functional analysis of the 4,774 PacBio-isoforms not present in SUGIT (Table S6), the top COG categories and the second-level GO terms (“molecular function” and “biological process” categories) are the same as for all PacBio-isoforms, whereas the most frequent term for the “cellular component” category (‘membrane’), is lower-ranked among all PacBio-isoforms. Considering third-level GO terms, the top “biological process”, “molecular function”, and “cellular component” are different from the top terms of all PacBio-isoforms (organic substance metabolic process, heterocyclic compound binding, cell part). The top three pathways (transporters, chromosome and associated proteins, and transfer RNA biogenesis) are also different from all PacBio-isoforms.

### Investigation of alternatively spliced transcripts

Although a complete sugarcane genome is not yet available, the sorghum genome can be used as a reference for sugarcane sequence analysis owing to the high gene identity between the two species (Jannoo et al., 2007). 72,061 PacBio-isoforms (60.38%) can be mapped to the sorghum genome, which is similar to the previous Iso-Seq study of sugarcane (69.4%) (Hoang et al., 2017). From BLAST results, 74,184 transcripts (68.03%) matched with sorghum transcripts annotated in the Phytozome database as the best hit. Out of these, 65,279 transcripts (88%) are highly similar to sorghum annotated gene sequences and can be mapped to the same genome location of the sorghum best hit genes. The remaining BLAST hits cannot be mapped to the sorghum genome, possibly because of low similarity to the hit gene, or other highly similar regions in the genome that make unique mapping difficult. The mapping statistics are summarized in Table S7.

**Figure 7:**
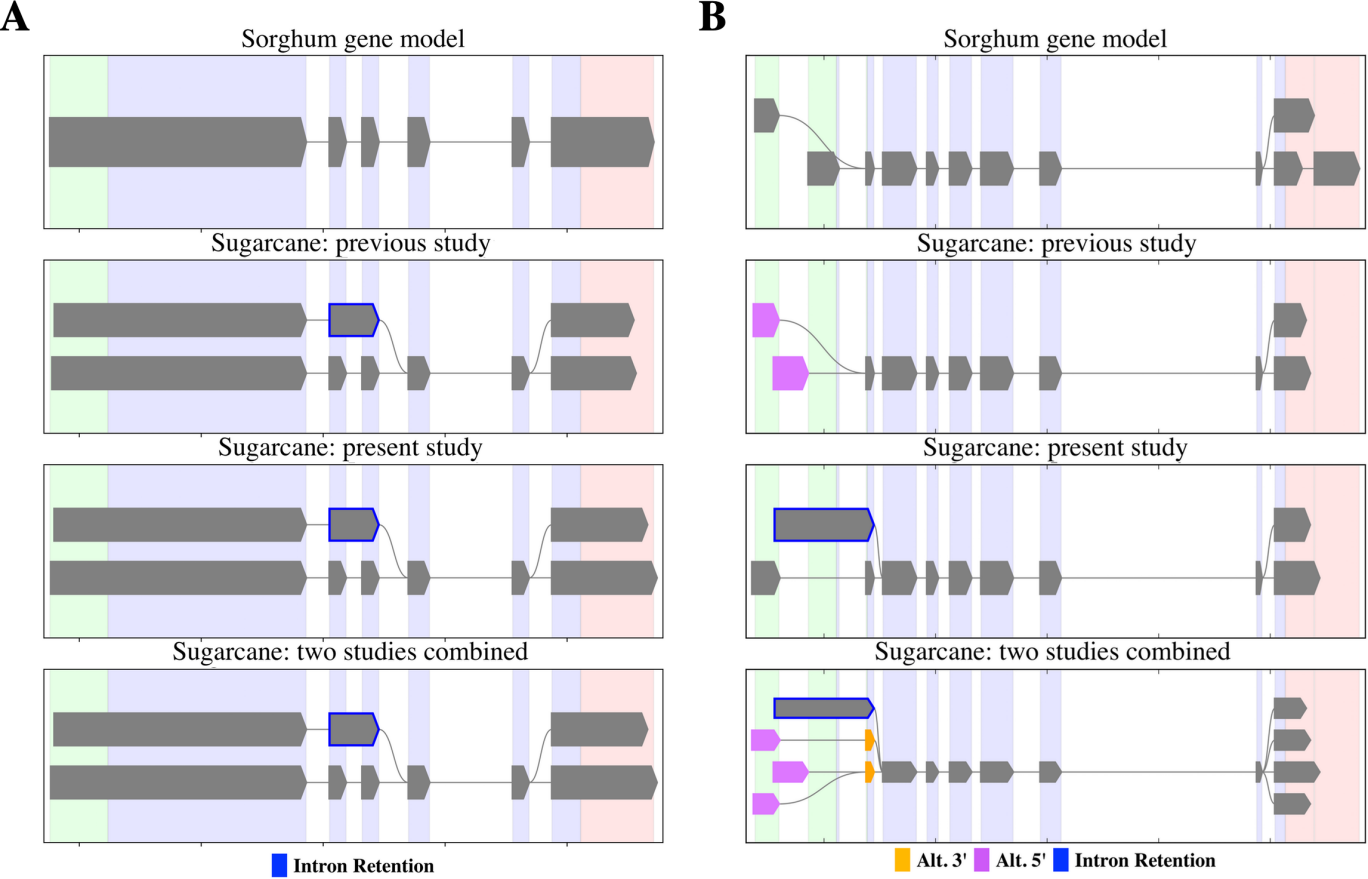
Alternative splicing patterns of PacBio transcripts. SpliceGrapher diagrams illustrate the splicing patterns of transcripts compared among the sorghum transcript annotation, the matched PacBio transcripts from the Hoang et al. (2017) study, the matched PacBio transcripts generated in the present study, and the matched PacBio transcripts combined from both studies. (A) Peptidase S24/S26A/S26B/S26C family protein (Sobic.002G223200) and (B) sucrose-phosphatase (Sobic.004G151800). Each color represents type of splicing event according to the data label.

PacBio-isoforms mapped to the sorghum genome were used to detect potential alternative splicing events represented among PacBio-isoforms. We identified 17,349 alternative splicing events comprising 5,410 intron retentions (31.2%), 1,423 skipped exons (8.2%), 5,695 alternative 3′ splice sites (32.8%) and 4,821 alternative 5′ splice sites (27.8%). The previous Iso-Seq study of sugarcane reported 4,870 alternative splicing events, in which alternative 3′ splice site was the most common and skipped exon the least common event (Hoang et al., 2017), the same pattern as for our data. We re-analyzed alternative splicing events represented among SUGIT sequences and found patterns of shared and distinct alternative transcripts between the two datasets, examples of which are shown in Fig. 7. For the first example, PacBio-isoforms from each dataset were mapped to the sorghum gene Sobic.002G223200 (peptidase S24/S26A/S26B/S26C family protein) (Fig. 7A). PacBio-isoforms from both datasets showed the same intron retention alternative splicing patterns, confirming the reproducibility of these sugarcane isoforms. Another example is shown for isoforms of the Sobic.004G151800 gene (sucrose-phosphatase) (Fig. 7B). Common alternative transcripts were found as shown by the combined results, whereas alternative transcripts unique to each study were also found.

### Analysis of transcriptome expression

The expression levels of PacBio transcript isoforms were explored based on the number of mapped Ion Proton reads which were obtained from the same leaf sample. The majority of transcripts have expression levels about 1-2 RPKM (Fig. S1). The numbers of transcripts with low (≥0.5 to 5 RPKM), medium (≥5 to 50 RPKM), and high (≥50 RPKM) expression levels are 50907, 17185, and 1868 transcripts, respectively. Since the lowly expressed genes are the major population, COG analysis of this group (Fig. S1) showed the same top three categories (signal transduction mechanism, general function prediction only, and posttranslation modification, protein turnover, chaperones) as identified in the analysis of all transcripts (“Functional annotation of protein-coding transcripts” subsection). The medium expressed group showed the similar trend to lowly expressed group with the ranks switching between ‘signal transduction mechanism’ and ‘general function prediction only’. The highly expressed genes tend to have different functions in which the top three COG categories are posttranslation modification, protein turnover, chaperones (26.95%), carbohydrate transport and metabolism (10.68%), and translation, ribosomal structure and biogenesis (9.11%). These highly expressed genes were found to be involved in several pathways including photosynthesis, pyruvate metabolism, nitrogen metabolism, ubiquitin system, glycolysis/gluconeogenesis, and RNA transport (Table S8 ).

## Discussion

The PacBio-isoforms reported here represent the first transcript isoform dataset of the KK3 sugarcane cultivar. We used the Iso-Seq method for exploring the transcriptomic complexity of this cultivar, in particular long transcripts. KK3 PacBio-isoforms are substantially longer (N50 = 3,611 bp) than previous sugarcane transcriptomic studies employing second-generation sequencing platforms, in which the N50 ranged from 640–1,385 bp (Cardoso-Silva et al., 2014; Dharshini et al., 2016; Huang et al., 2016; Li et al., 2016; Nishiyama Jr et al., 2014; Schaker et al., 2016; Vicentini et al., 2015). The high complexity of the sugarcane transcriptome hinders *de novo* assembly of long transcripts from short-read data, such that longer transcripts are under-represented. The N50 of KK3 PacBio-isoforms is also greater than the previous Iso-Seq study of sugarcane (Hoang et al., 2017). The transcript normalization procedure employed in that study, together with the different size-selection procedure may be the main reasons why the distributions of transcript lengths are substantially different between the Iso-Seq datasets.

By comparison of nucleotide and protein sequences with sugarcane and plant databases, we annotated the functions of most PacBio-isoforms. Although some unannotated sequences appear to be full-length and contain complete ORFs, the majority of unannotated sequences lacking an ORF longer than 300 bp have partial match with known protein sequences and may represent truncated cDNAs. This is borne out by the low average PacBio transcript coverage of about 23.94% and hit protein coverage of about 43.10%. Truncated cDNA molecules can acquire sequencing adapters in the Iso-Seq method and be erroneously assigned as full-length transcripts (Cartolano et al., 2016). In addition to truncated cDNA, the Iso-Seq protocol is prone to other experimental artifacts including spurious antisense transcripts (Tardaguila et al., 2018). We performed length comparison of PacBio-isoforms with matched sequences in other databases by selecting only hits in the sense direction to exclude confounding by antisense artifacts. Some of the 201 novel PacBio-isoforms with no matching database sequence could also be experimental artifacts, and alignment to a complete sugarcane reference genome sequence is required for determining whether they are genuine transcripts, in particular the putative lncRNAs.

Given that most PacBio-isoforms match with annotated sugarcane sequences, it is not unexpected that the annotation patterns of PacBio-isoforms are similar to previous studies of sugarcane. Protein kinase was identified as the second most common domain in this study, whereas this domain was the most common in a previous study (Nishiyama Jr et al., 2014). The top three COG categories represented by PacBio-isoforms are “signal transduction mechanism”, “general function prediction only” and “posttranslational modification, protein turnover and chaperones”, which are also the top three COG categories in sorghum (Fig. 4). A previous sugarcane study identified “replication, recombination and repair” as the top COG category (Cardoso-Silva et al., 2014) with 20.49% found, while only 1.46% was identified in this category for our dataset. The difference in representation of protein domains and COG categories between studies is possibly due to the different protein domain databases used. The representations of different annotation labels may also differ among transcriptome databases because of differences in transcript abundance. Transcripts functioning in signal transduction may be relatively more abundant in the KK3 cultivar sample we used than the pooled leaf sample from six sugarcane cultivars employed by (Cardoso-Silva et al., 2014).

The PacBio-isoforms matched with more sequences in the SUGIT database (Hoang et al., 2017) than any other sugarcane database as expected, since the same sequencing platform was used. The high degree of overlap is reflected by the same patterns of top GO terms among annotated functions and the same patterns of repetitive elements embedded in the transcripts. On the contrary, the PacBio-isoforms in this study complement the SUGIT database by providing novel transcripts in addition to greater representation of long and full-length transcripts. More data from different tissues and developmental stages are needed in order to provide a reference transcriptome for the KK3 cultivar. However, a comprehensive isoform catalog will require complete, annotated sugarcane reference genomes for sequence comparison.

## Conclusions

We generated a sugarcane leaf transcriptome dataset of the KK3 cultivar using the long-read PacBio SMRT sequencing platform. Transcripts from this dataset are on average, longer and more representative of full-length transcripts than reported in previous sugarcane transcriptome studies. Our data provide information of full-length transcripts which can assist researchers to understand more about sugarcane gene structure and facilitate sugarcane research including genome assembly and annotation, SNP identification, and molecular marker development for breeding programs in the future.

##  Supplemental Information

10.7717/peerj.5818/supp-1 Table S1 Summary of sugarcane Iso-Seq data and analysis results

10.7717/peerj.5818/supp-2 Table S2 Newly identified sugarcane transcripts The details of 5,026 PacBio transcripts which do not match with sugarcane sequences available in public databases are shown including sequence length, predicted ORF length, BLAST results against protein databases, the presence of protein domain(s), similarity with known long-noncoding RNA, gene expression level, and RepeatMasker analysis. 569 transcripts which do not match with protein databases are highlighted in green.

10.7717/peerj.5818/supp-3 Table S3 Predicted protein domains of ORFs The details of protein domains/signatures identified on PacBio transcripts.

10.7717/peerj.5818/supp-4 Table S4 Putative new lncRNA transcripts 1,216 PacBio-isoforms assigned as putatively novel lncRNAs are shown along with the details of ORF length (if any), expression levels, and repeat analysis.

10.7717/peerj.5818/supp-5 Table S5 GO, KOG, and KEGG pathway annotation results The number of mapped transcripts are shown for each GO, KOG, and KEGG category.

10.7717/peerj.5818/supp-6 Table S6 PacBio transcripts uniquely identified in this study The list of 4,774 PacBio transcripts generated in this study which do not match with PacBio transcripts generated in the Hoang et al. study were identified based on BLAST similarity search with *E*-value cutoff of 10^−20^. The details of these transcripts shown include sequence length, predicted ORF length, BLAST results against various databases, the presence of protein domain, similarity with known long-noncoding RNA, gene expression level, RepeatMasker analysis, and functional analysis (GO, KOG, and KEGG annotation).

10.7717/peerj.5818/supp-7 Table S7 Mapping statistics of sugarcane PacBio sequences to sorghum genome

10.7717/peerj.5818/supp-8 Table S8 Highly expressed PacBio transcripts 1,868 PacBio-isoforms generated in this study which show expression level ≥ 50 RPKM are shown along with the details of expression levels and functional analysis.

10.7717/peerj.5818/supp-9 Figure S1 Transcriptome expression analysis of PacBio transcripts (A) Frequency distribution of PacBio transcript expression levels. (B) Distribution of KOG functional classes identified in three transcript groups with high, medium, and low expression levels.

## Additional Information and Declarations

### Data Availability

The following information was supplied regarding data availability: PacBio and Ion Proton raw read data were deposited in the NCBI Sequence Read Archive (SRA) database under accession number SRP103465.
