# Supplementary material for: Uncovering full-length transcript isoforms of sugarcane cultivar Khon Kaen 3 using single-molecule long-read sequencing
Source: PeerJ. 2018 Oct 30;6:e5818. doi: 10.7717/peerj.5818 (PMC6214230; doi:10.7717/peerj.5818)
Supplement: Table S1 [file peerj-06-5818-s001.docx]

|  | Size fraction | | | |
| --- | --- | --- | --- | --- |
|  | 1-2 kb | 2-3 kb | 3-6 kb | 5-10 kb |
| Number of reads of insert | 1,881,202 | 1,235,225 | 800,717 | 242,030 |
| - N50 | 1,662 | 2,693 | 3,998 | 5,998 |
| - min-max length | 35-44,787 | 35-43,548 | 35-40,899 | 35-40,070 |
| Number of full-length, non-chimeric reads | 70,744 | 76,707 | 62,274 | 22,603 |
| - N50 | 1,407 | 2,469 | 3,646 | 5,705 |
| - min-max length | 302-17,963 | 334-17,248 | 471-26,595 | 381-20,683 |
| Number of polished high-quality isoforms | 19,907 | 21,023 | 10,974 | 2,095 |
| - N50 | 1,419 | 2,459 | 3,614 | 5,592 |
| - min-max length | 307-2,404 | 338-5,577 | 2,953-8,210 | 381-7,084 |
| Total number of polished high-quality isoforms (all size ranges) | 53,999 | | | |
| Number of polished low-quality isoforms | 15,921 | 20,697 | 25,571 | 13,786 |
| - N50 | 1,528 | 2,606 | 3,815 | 5,841 |
| - min-max length | 332-17,963 | 334-17,248 | 473-26,595 | 523-20,683 |
| Total number of polished low-quality isoforms (all size ranges) | 75,975 | | | |
| Total number of polished isoforms (all size ranges) | 129,974 | | | |
| Number of non-redundant isoforms after short-read error correction | 119,339 (length 307-26,751 bp, N50 = 3,611) | | | |
