# Supplementary material for: Uncovering full-length transcript isoforms of sugarcane cultivar Khon Kaen 3 using single-molecule long-read sequencing
Source: PeerJ. 2018 Oct 30;6:e5818. doi: 10.7717/peerj.5818 (PMC6214230; doi:10.7717/peerj.5818)
Supplement: Table S7 [file peerj-06-5818-s007.docx]

| PacBio transcripts  **[119,339]** | Mapped  **[72,061]** | BLAST hit to sorghum  **[66,254]** | - Mapped to same gene from BLAST: **65,279**  - Mapped to different gene from BLAST: **975**  - Mapped to intergenic region: **407** |
| --- | --- | --- | --- |
|  |  | BLAST hit to other plants  **[4,007]** | - Mapped to gene region: **3,875**  - Mapped to intergenic region: **132** |
|  |  | No BLAST hit  **[33]** |  |
|  | Unmapped  **[47,278]** | BLAST hit to sorghum  **[32,083]** |  |
|  |  | BlAST hit to other plants  **[15,195]** |  |
|  |  | No BLAST hit  **[4,556]** |  |
