# Supplementary figures and images for: Uncovering full-length transcript isoforms of sugarcane cultivar Khon Kaen 3 using single-molecule long-read sequencing
Source: PeerJ. 2018 Oct 30;6:e5818. doi: 10.7717/peerj.5818 (PMC6214230; doi:10.7717/peerj.5818)

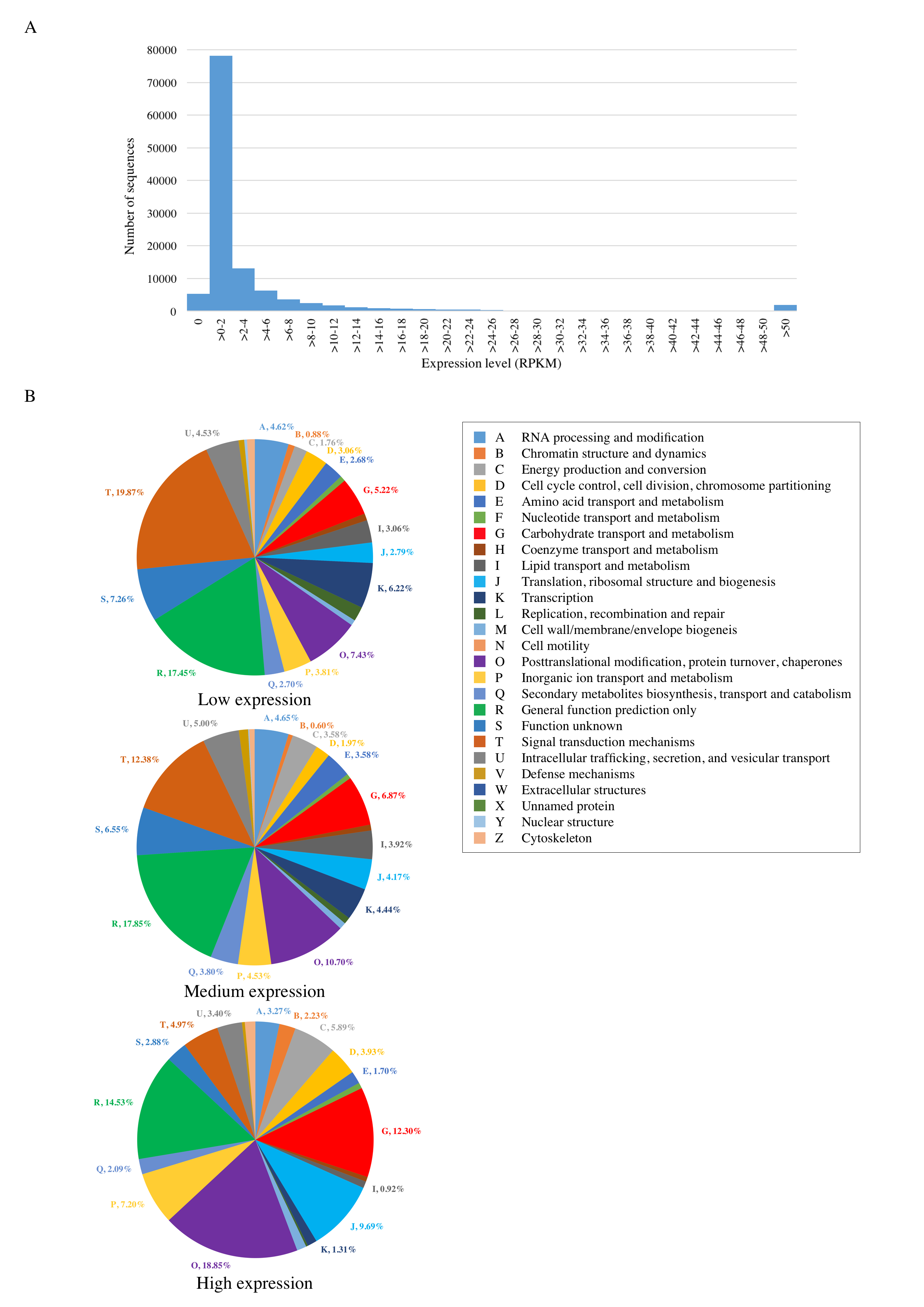

Supplement: Figure S1 — (A) Frequency distribution of PacBio transcript expression levels. (B) Distribution of KOG functional classes identified in three transcript groups with high, medium, and low expression levels. [file peerj-06-5818-s009.png]
